# Anti-inflammatory, antiproliferative and cytoprotective potential of the *Attalea phalerata* Mart. ex Spreng. pulp oil

**DOI:** 10.1371/journal.pone.0195678

**Published:** 2018-04-10

**Authors:** Fernando Freitas de Lima, Caroline Honaiser Lescano, Jucicléia da Silva Arrigo, Cláudia Andrea Lima Cardoso, Janclei Pereira Coutinho, Iluska Senna Bonfá Moslaves, Thalita Vieira do Nascimento Ximenes, Monica Cristina Toffoli Kadri, Simone Schneider Weber, Renata Trentin Perdomo, Cândida Aparecida Leite Kassuya, Maria do Carmo Vieira, Eliana Janet Sanjinez-Argandoña

**Affiliations:** 1 Federal University of Grande Dourados, Faculty of Health Sciences, Dourados, MS, Brazil; 2 University of Campinas, Faculty of Medical Sciences, Campinas, SP, Brazil; 3 State University of Mato Grosso do Sul, Course of Chemistry, Dourados, MS, Brazil; 4 University of Campinas, Faculty of Food Engineering, Campinas, SP, Brazil; 5 Federal University of Mato Grosso do Sul, Center of Biological and Health Sciences, Campo Grande, MS, Brazil; 6 Federal University of Grande Dourados, Faculty of Agricultural Sciences, Dourados, MS, Brazil; 7 Federal University of Grande Dourados, Faculty of Engineering, Dourados, MS, Brazil; Cairo University Faculty of Pharmacy, EGYPT

## Abstract

The anti-inflammatory, antiproliferative and cytoprotective activity of the *Attalea phalerata* Mart. ex Spreng pulp oil was evaluated by *in vitro* and *in vivo* methods. As for the chemical profile, the antioxidant activity was performed by spectrophotometry, and the profile of carotenoids and amino acids by chromatography. Our data demonstrated that *A*. *phalerata* oil has high carotenoid content, antioxidant activity and the presence of 5 essential amino acids. In the *in vitro* models of inflammation, the oil demonstrated the capacity to inhibit COX1 and COX2 enzymes, the production of nitric oxide and also induces macrophages to spreading. In the *in vivo* models of inflammation, the oil inhibited edema and leukocyte migration in the *Wistar* rats. In the *in vitro* model of antiproliferative and cytoprotective activity, the oil was shown inactive against the kidney carcinoma and prostate carcinoma lineage cells and with cytoprotective capacity in murine fibroblast cells, inhibiting the cytotoxic action of doxorubicin. Therefore, it is concluded that *A*. *phalerata* pulp oil has anti-inflammatory effects with nutraceutical properties potential due to the rich composition. Moreover, the oil also has cytoprotective activity probably because of its ability to inhibit the action of free radicals.

## Introduction

The fruits of *Attalea phalerata* Mart. ex Spreng. (Arecaceae) are known as bacuri, acuri or motacu in the Brazilian Cerrado. The pulp of "bacurizeiro" fruits are important foods for animals such as agouti, wild boars, macaws, parakeets, besides serving as raw material for starch development [1,2]. The high nutritional value of the fruit presents a promising nutraceutical food due to the presence of beneficial minerals, high amount of lipids, most of which are short chain saturated fatty acids and unsaturated fatty acids in addition to the presence of provitamin A carotenoids [1,3,4].

The pulp oil is popularly used to relieve joint pain, pulmonary congestion [1,2] and antipyretic [5]. Additionally, it is applied as hair tonic against hair loss and treatment of dandruff [2]. The fruit has a high content of carotenoids in its composition, predominantly β-carotene, β-zeacarotene and α-carotene and also high levels of fatty acids, mainly monounsaturated [3,4,6].

Carotenoids are antioxidants that assist in the maintenance of the immune system, to prevent chronic disease [6–9] and regulation of inflammatory processes by inhibiting the expression of nitric oxide synthase (iNOS) and cyclooxygenase (COX1 and COX2) [10,11]. In tumorigenesis, carotenoids are able to protect the human body from cellular damage caused by oxidative stress and decrease the risk of mutations through their ability to inactivate free radicals [9,12]. The prevention process takes place by direct action in free radicals in order to avoid oxidative stress and DNA damage in the human body [13]. Despite the occurrence of antioxidants endogenous production, it is recommended to complement with antioxidants from food sources, such as carotenoid supplementation from natural products [14,15].

According to the above, the objective of the present study was to evaluate the anti-inflammatory, antiproliferative and cytoprotection effect of the bioactive compounds present in *A*. *phalerata* Mart pulp oil.

## Material and methods

### Plant material and oil extraction

The fruits of *A*. *phalerata* were collected in the municipality of Rio Brilhante–MS (21° 48'07 "S 54° 32'47" W). The voucher specimen of the species was deposited in the Herbarium of the Universidade Federal da Grande Dourados (UFGD) No. DDMS5033. The pulp of the fruit was dehydrated to obtain the oil, which was carried out with 1:3 hexane solvent (pulp: solvent, w/v) under continuous stirring (150 rpm) for 7 days at room temperature in the dark. After extraction, for complete removal of the solid residue, the material was filtered and the solvent was completely removed under reduced pressure at 40°C in a rotary evaporator. The *A*. *phalerata* oil pulp (APOP) was packed in an amber bottle and refrigerated at 9°C until the beginning of the analysis.

### Analytical evaluation

#### Analysis of carotenoids and amino acids by high performance liquid chromatography

The sample was analyzed in a high-performance liquid chromatography (HPLC) analytical system (LC-56AD, Shimadzu, Kyoto, Japan) with a binary solvent using ODSHYPERSIL column (C-18, 150 mm long x 4.6 mm of Internal diameter, 5 μm particle size, Thermo Electron Corporation) and diode array detector (PAD) monitored at *λ* = 200–800 nm. All chromatographic analyzes were performed at 22°C.

Carotenoids: Elution was started using 90% acetonitrile, 10% ethyl acetate, in 15 min 50% acetonitrile, 50% ethyl acetate and in 25 min returning to the initial condition. The flow rate and volume injected were 0.7 mL min^-1^ and 20 mL, respectively. The identification of the compounds was based on the absorption spectra in the UV region and associated with standards for α-carotene and β-carotene. Carotenoid quantification in the APOP was performed by external calibration curve. The concentrations of α-carotene and β-carotene were determined with the respective standards. Due to the lack of analytical standards, the amount of ζ-carotene was determined using the β-carotene curve.

Amino acids: Eluent A consists of a solution of 25 mM acetic acid and 0.02% sodium azide in ultrapure water, adjusted to pH 6 and the eluent B consists of acetonitrile. Elution was started using 96% of eluent A, 4% of eluent B, in 30 min 69% of eluent A, 31% eluent B and in 40 min returning to the initial condition. The flow rate and volume injected were 0.9 mL min^-1^ and 20 μL, respectively. The identification of the compounds was based on absorption spectra in the UV region and associated to standards for alanine, arginine, isoleucine, methionine, proline, serine, threonine, tryptophan and valine. The amino acid quantification in APOP was performed by external calibration curve and the concentration of alanine, arginine, isoleucine, methionine, proline, serine, threonine, tryptophan and valine were determined with respective standards.

A linear regression (or least squares) of the peak areas as a function of the concentrations was performed to determine the correlation coefficients. The equation parameters (slope and intercept) of the standard curve were used to obtain the concentration values for the samples.

#### Evaluation of the antioxidant capacity by the β-carotene / linoleic acid system

The antioxidant capacity of the β-carotene / linoleic acid system was adapted from Marco [16] and Dos Santos et al. [17]. Firstly, the β-carotene / linoleic acid solution was prepared by dissolving 40 μL of linoleic acid, 530 μL of Tween 40, 50 μL of the β-carotene solution and, to complete the solubilization, 1 mL of chloroform. After complete solubilization of the compounds, the chloroform was evaporated, and oxygenated distilled water was slowly added to the flask with vigorous agitation. The samples were prepared using 5 mL of the solution of the β-carotene / linoleic acid system, 400 μL of Trolox (50 μg mL^-1^ to 800 μg mL^-1^) and 400 μL of APOP extract at different concentrations (50 μg mL^-1^ to 800 μg mL^-1^), homogenized and taken to the water bath at 40°C for 120 min. Spectrophotometric readings (Biochrom® Libra S60) were performed every 15 min for 120 min at 470 nm. All determinations were performed in triplicate. The antioxidant activity of the oil was expressed as % of inhibition of oxidation ± standard deviation.

### Animals

Adult female *Wistar* rats (200–250 g) and adult male *Swiss* mice (30–50 g) were used, from the Central Animal House of the Universidade Federal da Grande Dourados (UFGD). The animals were kept in collective cages (5 animals/cage) at controlled temperature (22 ± 1°C), light cycle (12 h light / dark), treated *ad libitum* water and commercial rodent feed. At the end of the experiments, the euthanasia of the animals was performed with isoflurane overdose followed by cardiac puncture exsanguination for the rats and CO_2_ chamber for the mice. The experiments were conducted according to the standards of the Conselho Nacional de Controle de Experimentação Animal (CONCEA) and the tests were previously approved by the Comissão de Ética no Uso de Animais (CEUA-UFGD) (protocols 21–2013 and 22–2015).

### *In vitro* anti-inflammatory evaluation

#### Obtainment of murine peritoneal macrophages

The mice were pretreated with 1.5 mL of a 3% thioglycolate solution, intraperitoneally (ip), 96 h prior to collection of the cells. After this period, they were submitted to euthanasia and macrophages obtained by washing the peritoneal cavity with 3 mL of buffered saline (PBS) in a sterile laminar flow chamber. The lavage from peritoneal cavity was centrifuged (700 rpm/5 min) and the supernatant discarded. The cells were resuspended in 1 mL of RPMI 1640 culture medium. Then, the total count was carried out determining the number of mL^-1^ cells. From this, dilutions were performed to obtain 2x10^6^ mL^-1^ cells. The peritoneal macrophages obtained were used in tests 2.4.2., 2.4.3. and 2.4.4.

#### Cell viability by the MTT assay

Cell viability is determined by the MTT (3- (4,5-dimethylthiazol-2yl) -2,5-diphenyl tetrazoline bromide) assay described by Mosmann [18]. Peritoneal macrophages were distributed in 96 well plates (2x10^5^ cells/well) and incubated at 37°C in 5% CO_2_ atmosphere. After 24 h the cells were treated with different concentrations of APOP (1, 5 and 10 mg mL^-1^) for 16 h in RPMI 1640 culture medium. Then, the MTT (20 mL, 5 mg mL^-1^) was added to each well and the cells incubated for 2 h. After formation of the resulting crystals from the cells, it was dissolved in DMSO (dimethylsulfoxide, 200 μL). The absorbances were determined using an ELISA microplate reader (Anthos Labtec® LP 400) at a wavelength of 540 nm.

#### Production of nitric oxide (NO) by peritoneal macrophages

For determination of NO production the macrophage suspension (2x10^5^ cells 100 μL^-1^) was incubated for 60 min in the 96-well plate in RPMI 1640 medium at 37°C in 5% CO_2_ atmosphere for adhesion. The non-adherent cells were removed after this period. LPS (*E*. *coli* lipopolysaccharide, 1 μg mL^-1^, 10 μL) and/or APOP (1 mg mL^-1^, 10 μL) were diluted in RPMI 1640 and added to the plate. Cells were incubated for 48 h, and at the end of this period, the production of NO was determined by the accumulation of nitrite in the supernatants of the cell culture, which was quantified by the Griess method [19]. Aliquots of the supernatants were added to an equal volume of Griess reagent [1% sulfanilamide / 0.1% n-(1-naphthyl) in 2.5% phosphoric acid) and incubated for 10 min at room temperature. Then, the absorbance was determined on an ELISA sensor (Anthos Labtec® LP 400) at wavelength of 540 nm. The nitrite concentration values were extrapolated from a calibration curve of sodium nitrite as standard and expressed as μM of NO_-2_.

#### Spreading of macrophages

The macrophage spreading assay was performed according to the methodology described by Rabinovitch and DeStefano [20]. Peritoneal macrophages (2x10^5^ cells, 100 μL^-1^) were dispersed on glass coverslips in a 24-well plate and incubated with 1 mL of RPMI 1640 medium in the presence or absence of APOP (10 μL, 1 mg mL^-1^) and/or LPS (10 μL, 1 μg mL^-1^) at 37°C in 5% CO_2_ atmosphere. Subsequently, the coverslips were washed with PBS and the cells adhered to the glass were fixed with 2.5% glutaraldehyde and examined in a phase contrast microscope (Optphase®) at magnification of 400x. One hundred macrophages were counted and classified as spread or not, according to morphological criteria. Spread macrophages index was presented as percentage of spread macrophages related to the count of 100 cells.

#### COX1 and COX2 inhibition

The inhibition assay of cyclooxygenase 1 (COX1) and cyclooxygenase (COX2) was performed using three distinct groups, with diclofenac sodium as the reference compound. The assay included both ovine COX1 enzymes and recombinant human COX2 enzymes to perform the screening of specific inhibitors using diclofenac sodium as standard. The compounds tested were added to both COX1 and COX2 with the substrates and buffers to perform the COX reaction according to instructions from the COX Inhibition Assay Kit (Item No. 560131, Cayman Chemicals Company, USA). Compounds were incubated with the enzymes for 10 min at 37°C and absorbance was read using a plate reader (Synergy H1 Hibrid Reader-Biotek) at 412 nm.

### *In vivo* anti-inflammatory evaluation

#### Carrageenan-induced paw edema

In the *in viv*o evaluation for Carrageenan-induced paw edema *Wistar* rats were randomly distributed in five groups, 5 animals/group. Group 1 (negative control) was treated by oral means with vehicle (0.9% saline); Groups 2, 3 and 4 were given 300, 500 and 700 mg kg^-1^ of APOP, respectively. The doses used in the study were stipulated in pre-tests and established under the non-toxic limits of daily doses [4]. After one hour, the animals received 50 μL of 0.9% saline containing 300 μg of carrageenan in the right hind paw. The same volume of saline solution was given to the left hind paw. The edema was evaluated in both paws 0.5; 1; 2 and 4 h after the injection of carrageenan, with the assistance of a digital plethysmometer [21,22].

#### Pleurisy induced by carrageenan

In the *in vivo* evaluation by the experimental model of pleurisy induced by carrageenan, *Wistar* rats were randomly assigned to four groups, 5 animals/group. Group 1 (naive), which did not receive intrapleural carrageenan injection; Group 2 (negative control) was treated with vehicle (0.9% saline solution); In group 3, dexamethasone (1 mg kg^-1^, subcutaneously) was applied and group 4 was treated with 700 mg kg^-1^ of APOP orally [4]. After 1h, pleurisy was induced in the experimental groups by intrapleural injection of 100 μL of carrageenan in 1% saline solution. The naive group received 100 μL of sterile saline by intrapleural injection. After 4 h, the animals were euthanized, and the pleural cavity was washed with 1 mL PBS. A 20 μL aliquot of the lavage (exudate) was collected from the pleural cavity and diluted in Turk's solution (1: 20) and the total leukocyte count was performed in a Neubauer chamber [23].

### Antiproliferative and cytoprotective evaluation

#### Antiproliferative evaluation

For the antiproliferative activity evaluation, two human neoplastic cell lines, 786–0 (ATCC-CRL-1932, kidney carcinoma), PC-03 (ATCC-CRL-1435, prostate carcinoma) and a normal cell line, NIH/3T3 (murine fibroblast) were used. Cells were incubated at 37°C (5% CO_2_) for extension and increase in cell density [24]. Samples (APOP and β-carotene) were resuspended in DMSO at the concentration of 0.1 g mL^-1^, diluted in complete culture medium and the highest dilution had a DMSO concentration of less than 0.4%. The test was performed with sulforhodamine B dye (SRB), which is based on its affinity for the basic proteins present in the intact cells fixed by trichloroacetic acid [25]. The test was performed with a T0 plate, where a complete medium and cell were placed, and test plate with triplicate of cell suspension, where the test samples were placed in concentrations of 0.25, 2.5, 25 and 250 μg mL^-1^. The T0 plate was incubated for 24 h at 37°C (5% CO_2_) and the test plate was incubated under the same conditions for 48 h after placing the test samples. The positive control was doxorubicin at concentrations of 0.025, 0.25, 2.5 and 25 μg mL^-1^. The result was obtained in absorbance at 540 nm and the dose for inhibition of 50% growth was determined.

#### Cytoprotective evaluation

NIH / 3T3 cells (7.5x10^3^cells/well) were grown in 96-well plates and treated with different concentrations of APOP (250, 500, 1000 and 2000 μg mL^-1^) in triplicate for 24 h at 37°C, 5% CO_2_ atmosphere. After 24 h, doxorubicin at the concentrations of 0.025, 0.25, 2.5 and 25 μg mL^-1^ was added to the wells with cell suspension and APOP, in increasing order of concentration, respectively for further 24 h. The percentages of growth were obtained as mentioned in the item antiproliferative activity.

### Statistical analysis

The results were expressed as mean ± standard deviation (SD) for the analytical evaluation experiments and standard error of the mean (SEM) for the biological experiments. The analysis of variance was performed with one-way ANOVA followed by the Tukey test to evaluate the possible differences between groups.

## Results and discussion

### Carotenoids, antioxidant activity and amino acids

Natural products are composed of numerous secondary metabolites, which perform particular actions in the human body, and can be beneficial or harmful. Important compounds are terpenes, carotenoids and phenolic compounds, which are associated with the fight against chronic diseases such as cancer, diabetes, heart disease, chronic inflammation and the fight against reactive oxygen and nitrogen species (ROS/RNS) [26–28].

The elucidation of the compounds contained in the natural products is of extreme importance for the knowledge of the mechanism of action of herbal medicines and nutraceutical foods. APOP is mainly composed of carotenoids, which are α-carotene (11.02 μg g^-1^ ± 0.3), β-carotene (62.33 μg g^-1^ ± 0.7) and ζ-carotene (1.94 μg g^-1^ ± 0.1) (Table 1). Studies point out that the different carotenoids play an important role in capturing free radicals by binding their structural chains, making them more stable [29,30].

**Table 1:** Analysis of carotenoids and amino acids in *A*. *phalerata* oil employing HPLC.

	Retention time (min)	Linear range (μg mL^-1^)	Intercept (a)	Slope (b)	Determination coefficient (R^2^)	Compound concentration (μg g^-1^ ± SD)
**Carotenoids**						
α-carotene	16.26	100–1000	3879.78	2980.99	0.994	11.02 ± 0.3
ζ-carotene	16.86	---	---	---	---	1.94 ± 0.1
β-carotene	18.15	100–1000	4237.56	9589.78	0.996	62.33 ± 0.7
**Amino acids**						
Serine	8.87	8–100	77158.69	2.70	0.997	1.18 ± 0.04
Threonine	10.98	8–100	10511.66	2.91	0.999	0.60 ± 0.01
Arginine	13.49	8–100	49917.22	2.61	0.998	0.25 ± 0.01
Alanine	14.26	8–100	42139.23	2.47	0.970	0.74 ± 0.02
Proline	16.66	8–100	12380.67	2.41	0.958	0.62 ± 0.01
Valine	24.99	8–100	29532.31	1.92	0.985	0.44 ±0.01
Methionine	25.87	8–100	6363.44	2.40	0.999	0.15± 0.01
Isoleucine	28.29	8–100	47234.52	2.01	0.983	0.28 ± 0.01
Tryptophan	28.88	8–100	10714.55	2.80	0.997	0.46 ± 0.01

ζ-carotene was determined employing curve of β-carotene. Values expressed in mean ± standard deviation (SD). Formule: y = a + bx, where y = ratio of peak areas, x = concentration (μg g^-1^), a = intercept and b = slope.

The carotenoids found in the oil may have had an influence on antioxidant activity by the β-carotene/linoleic acid method, with 47.33 ± 1% inhibition of oxidation after exposure of 120 min at a concentration of 200 μg mL^-1^, whereas Trolox (standard) showed inhibition of 61.64 ± 1% at a concentration of 200 μg mL^-1^ after the same exposure time. The method allowed to estimate the relative capacity of the antioxidant compounds contained in the APOP to inhibit the linoleic peroxide radicals from oxidizing the β-carotene contained in the system (β-carotene/linoleic acid emulsion) [31].

The oil is also composed of beneficial fatty acids, consisting of 20.61% of saturated fatty acids and 78.53% of unsaturated fatty acids; among these acids, 57.65% are monounsaturated and 20.88% polyunsaturated, predominantly oleic, linoleic and palmitic acids [4]. The unsaturated fatty acids, predominant in the oil of the fruit, when consumed of appropriate dose exert important function in the human organism, like the maintenance of the immune system in inflammatory processes [32,33] and reduction of body fat [34,35].

Complementary analysis by high-performance liquid chromatography identified the presence of nine amino acids (Table 1), which include five essential amino acids (isoleucine, methionine, threonine, tryptophan and valine). Serine was the amino acid found in highest concentration, followed in decreasing order of concentration by alanine, proline, threonine, tryptophan, valine, isoleucine, arginine and methionine. In the attempt to extract the carotenoids contained in the APOP, it was observed that the extraction method allowed the transition of the amino acids contained in the pulp to the oil, even in small concentrations.

Researchers have shown the presence of amino acids in Brazilian Cerrado fruits almonds and highlighted their nutritional importance in food [36,37]. Amino acids have an important functional role, as they perform the production of proteins, digestive enzymes and maintenance of muscles. They are responsible for the production of hormones and neurotransmitters [38–40].

#### *In vitro* anti-inflammatory evaluation

**Fig 1:**
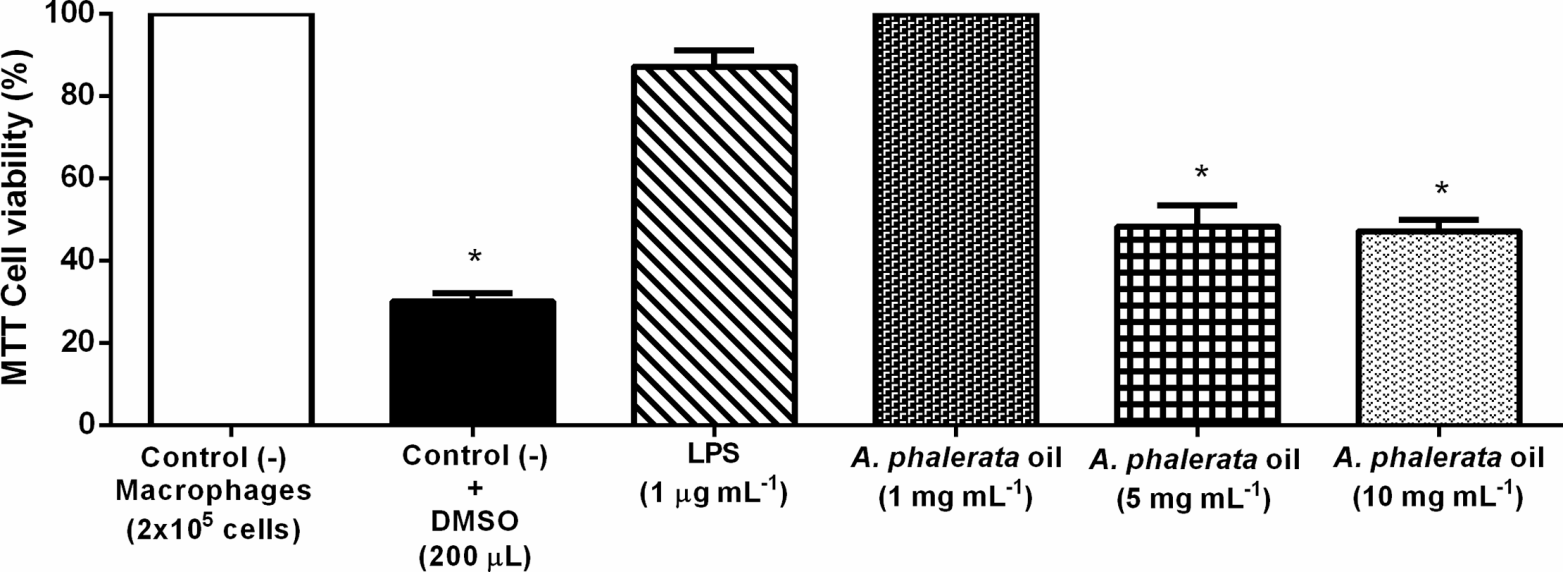
Effect of the *A*. *phalerata* oil in cell viability in macrophages cells by MTT assay. Values expressed in mean ± standard error of the mean. n = 3. * P <0.05 (ANOVA / Tukey) compared with the negative control (-). LPS–Lipopolysaccharide. DMSO–Dimethylsulfoxide.

Cell experiments have proven to be an important tool in elucidating the mechanism of action of natural substances and also serve as a prelude to *in vivo* testing. The MTT assay evaluates cell viability through the respiration of cells. APOP at 1 mg mL^-1^ did not influence cell viability, but doses of 5 mg mL^-1^ and 10 mg mL^-1^ were cytotoxic, reducing viability to approximately 50% (Fig 1).

A previous study with APOP in the same experimental model [3] did not present cytotoxicity in doses higher than that used in this study; However, the cells used were T84 (colon carcinoma). Thus, the difference between the results obtained can be attributed to the particularity of the cells in each study. Primary cells (macrophages from this study) present limited division capability and reach their senescence state more rapidly than immortalized cells, which are those obtained from collections of cell cultures and have their cell death capability deactivated [41–43].

**Fig 2:**
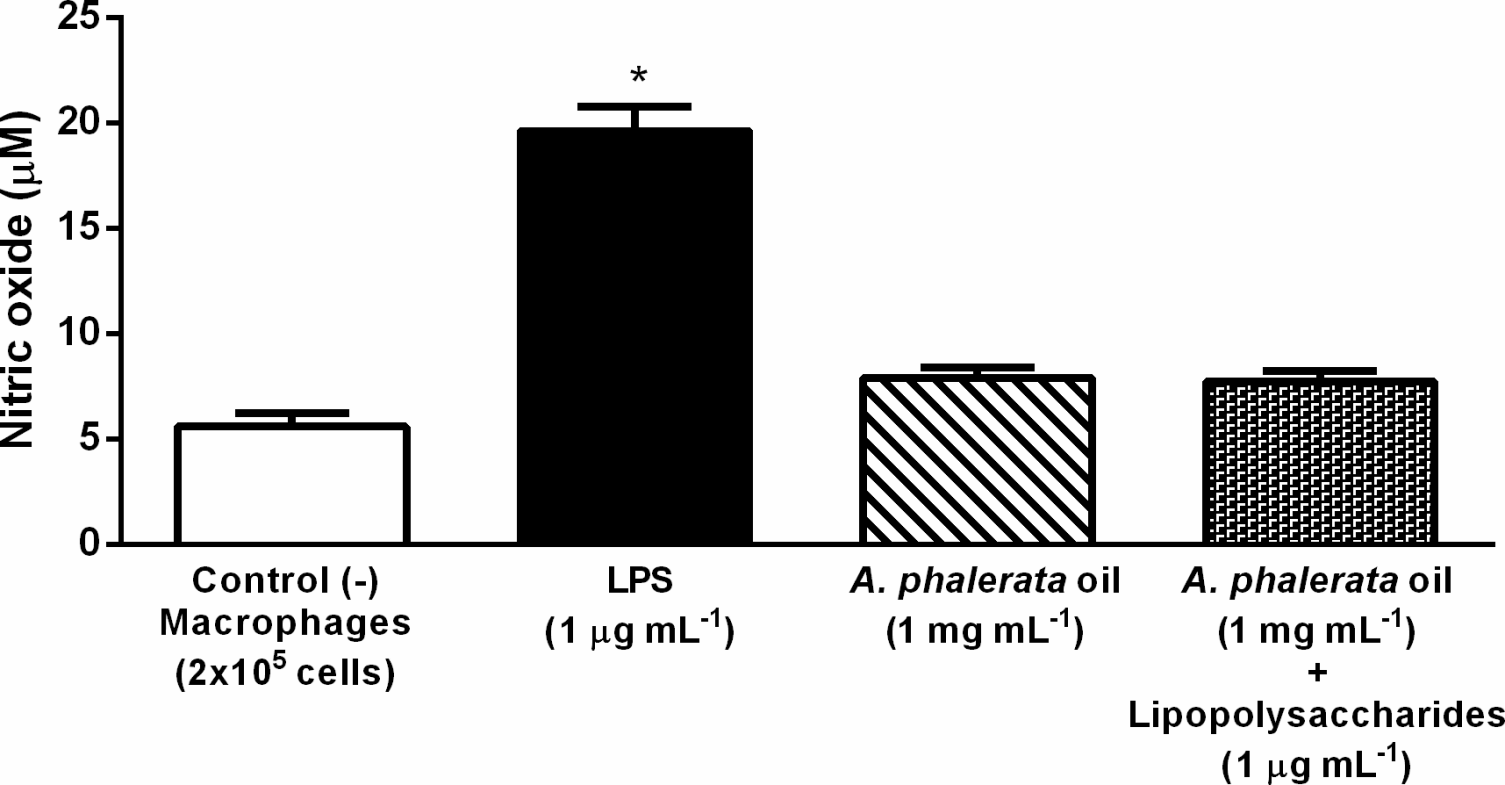
Effect of the *A*. *phalerata* oil in nitric oxide (NO) production. Values expressed in mean ± standard error of the mean. n = 33. * P <0.05 (ANOVA / Tukey) compared with the negative control (-). LPS–Lipopolysaccharide.

Although being important in the control of infectious diseases, due to its cytotoxicity, NO, when produced in an exacerbated way, presents clinical correlation with septic shock, autoimmune diseases, arteriosclerosis, tumorigenesis, genotoxicity and inflammation [44–46]. In this study, macrophages incubated with APOP (1 mg mL^-1^) did not show increase in NO release (Fig 2). Macrophages, when stimulated with LPS, produce NO, but this did not occur in the treatment of macrophages with APOP+LPS. Therefore, it is suggested that APOP, when acting in the production of NO, can act as an important cytoprotective/chemoprotective agent, modulator of tumorigenesis and acute inflammatory processes.

**Fig 3:**
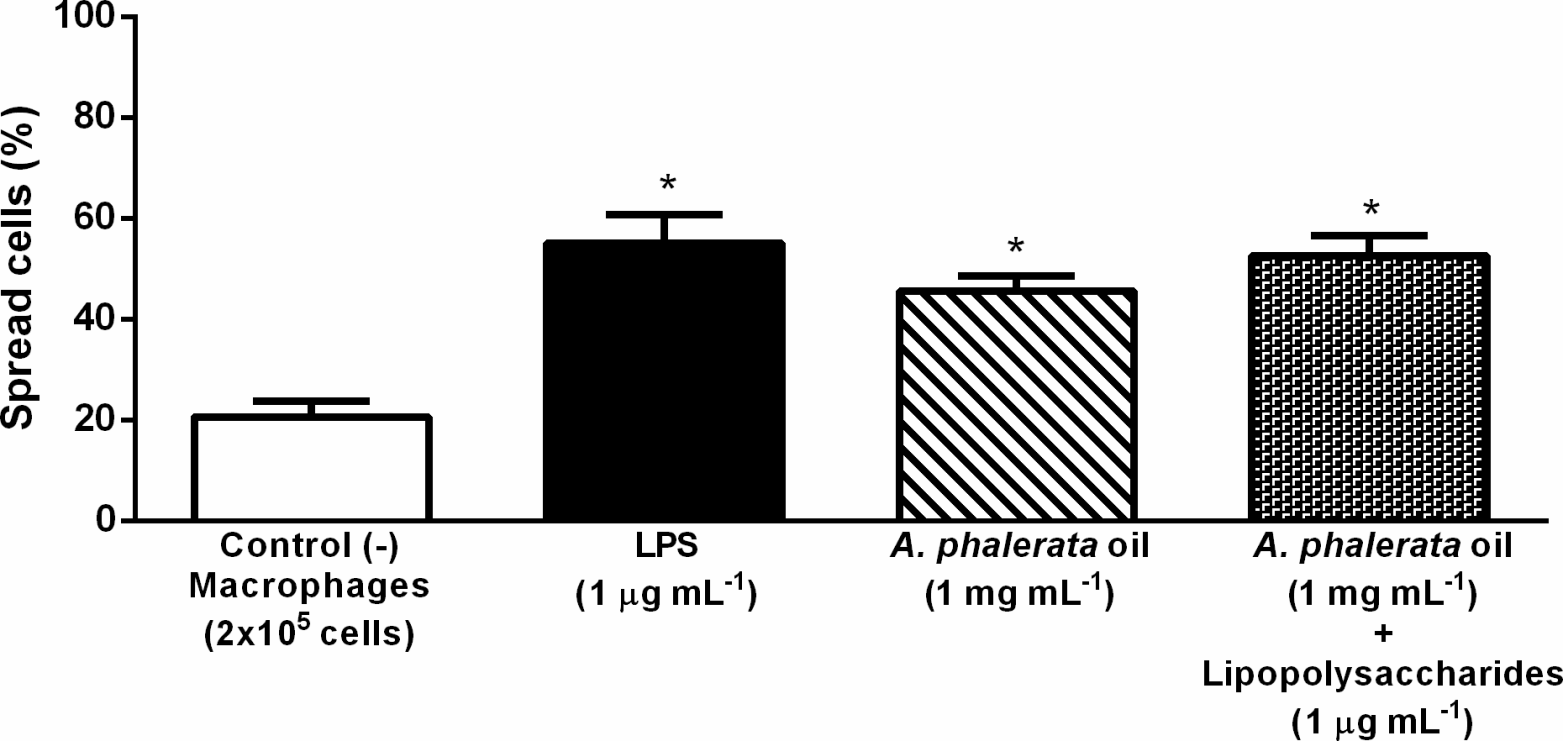
Effect of the *A*. *phalerata* oil in spreading of macrophages. Values expressed in mean ± standard error of the mean. n = 3. * P <0.05 (ANOVA / Tukey) compared with the negative control (-). LPS–Lipopolysaccharide.

The result of the macrophage spreading assay showed that APOP (1 mg mL^-1^) induced macrophage spreading. However, treatment with APOP did not affect LPS-induced spreading (Fig 3), while LPS induced macrophages to spreading as expected. The ability of the cells to spread can be considered a cellular activation index [20], predisposing the cells to the phagocytosis process [47,48].

The production of COX1 and COX2 enzymes occurs during inflammatory processes in the organism, where COX1 is found in several tissue cells and modulates physiological processes and COX2 is stimulated by invading organisms and proinflammatory cytokines [49], with the prostaglandins as final product. With the inhibition of the COX1 and COX2 enzymes, the production of prostaglandins is consequently inhibited, thus reducing the characteristic signs of inflammatory processes.

**Fig 4:**
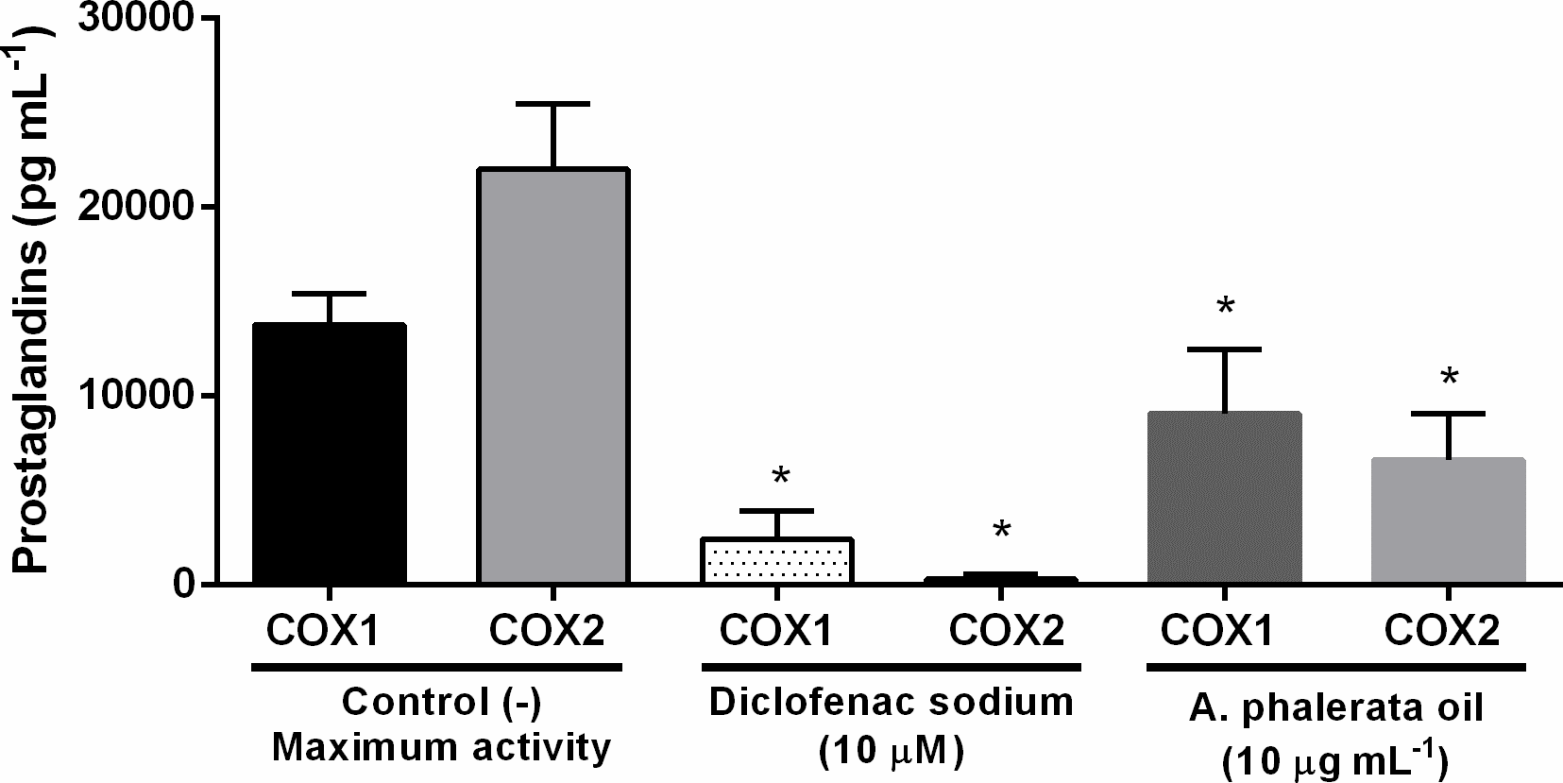
Effect of the *A*. *phalerata* oil in COX1 and COX2 inhibition. Values expressed in mean ± standard error of the mean. n = 4. * P <0.05 (ANOVA / Tukey) compared with the negative control (-).

Results for enzymatic inhibition of COX1 and COX2 (Fig 4) showed the inhibitory ability of APOP in both isoforms, which was 35% COX1 and 70% COX2, and did not differ statistically from the reference drug (diclofenac sodium). Therefore, the efficacy of inhibition of APOP (10 μg mL^-1^), used in folk medicine can be attributed to the suppression of the inflammatory response due to the ability to inhibit the activity of COX enzymes.

The high content of carotenoids presents in the APOP encouraged us to investigate the potential effect on the inhibition of COX; and production of nitric oxide, however, there are no data in the literature about its inhibition. The results found in the experiment of inhibition of COX1, COX2 and in the production of NO by macrophages corroborate with data published in the literature, where it is assumed that carotenoids act in the production of prostaglandins (inhibiting COX) and nitric oxide production, by inactivation of the NF-κB pathways [10,11].

### *In vivo* anti-inflammatory evaluation

The use of carrageenan is applied in several experimental models of inflammation and help in the development of anti-inflammatory drugs. The model of paw edema induced by carrageenan is a classic model of vascular inflammatory response related to the formation of edema [50]. The inflammatory response of carrageenan consists of three phases. The primary phase is mediated by histamine and serotonin, followed by the secondary phase mediated by endogenous non-peptidic bradykinin and the final phase mediated mainly by the production of prostaglandin E2 and induction of COX2 [51–53]. The results of the present study demonstrate that intraplantar injection increases the edema in the paw of the animals at times 0.5, 1, 2 and 4 h after the injection of carrageenan, corroborating with the results shown in the literature.

**Fig 5:**
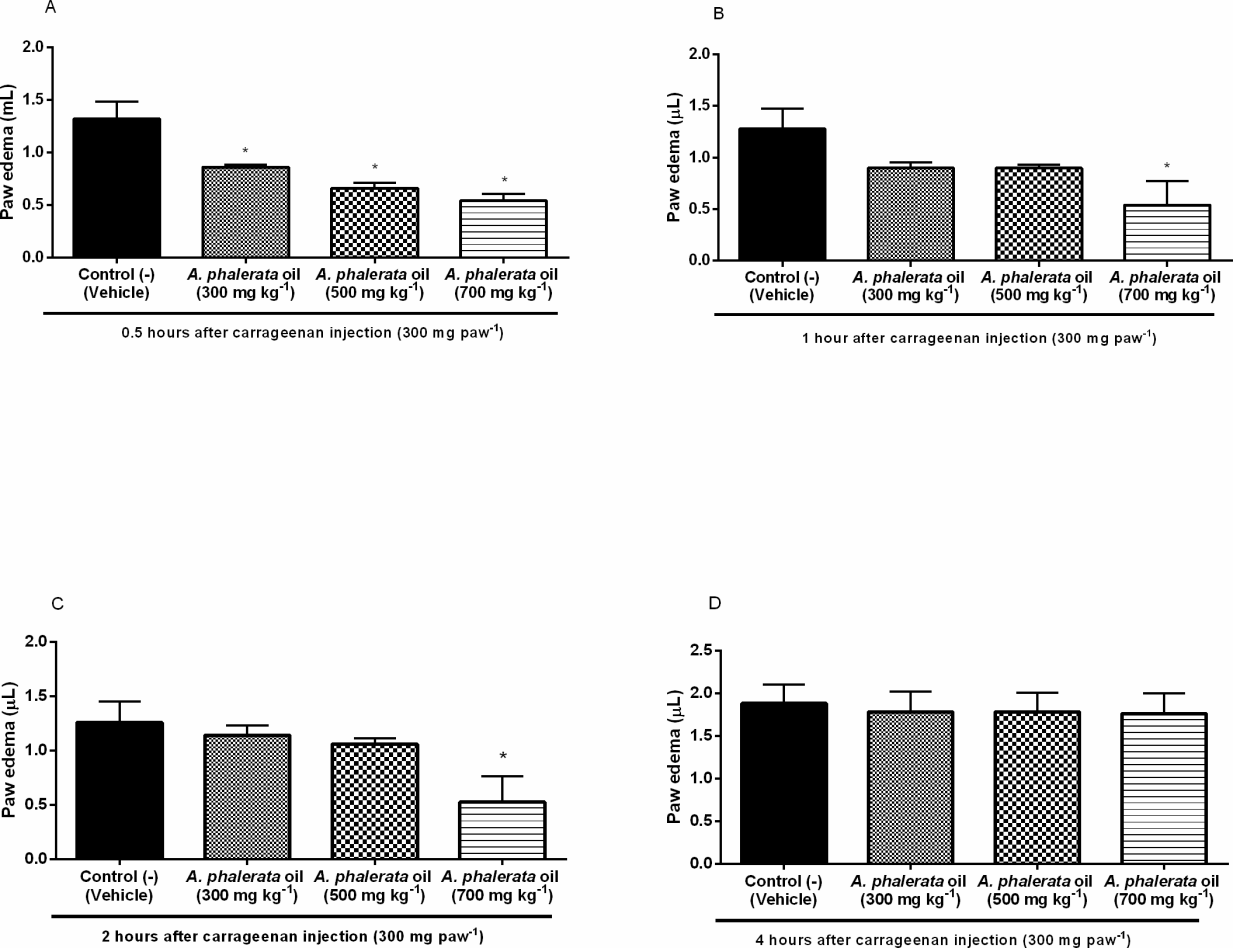
Effect of the *A*. *phalerata* oil on the paw edema after 0.5h (A), 1h (B), 2h (C) and 4h (D) of the intraplantar carrageenan injection. Values expressed in mean ± standard error of the mean. n = 5. * P <0.05 (ANOVA / Tukey) compared with the negative control (-).

Oral administration of the 700 mg kg^-1^ APOP dose significantly reduced edema after 0.5, 1 and 2 h of carrageenan injection. Treatment with APOP (700 mg kg-1) resulted in a 68 ± 7% reduction of edema after 0.5 h of treatment (Fig 5A) and inhibition of edema remained significant after 1 h (Fig 5B) and after 2 h (Fig 5C). It is suggested that APOP performs activity at all stages of Carrageenan-induced edema formation, and therefore has action under the chemical mediator’s histamine, serotonin, bradykinin, prostaglandin E2 and COX, and non-specific effect.

**Fig 6:**
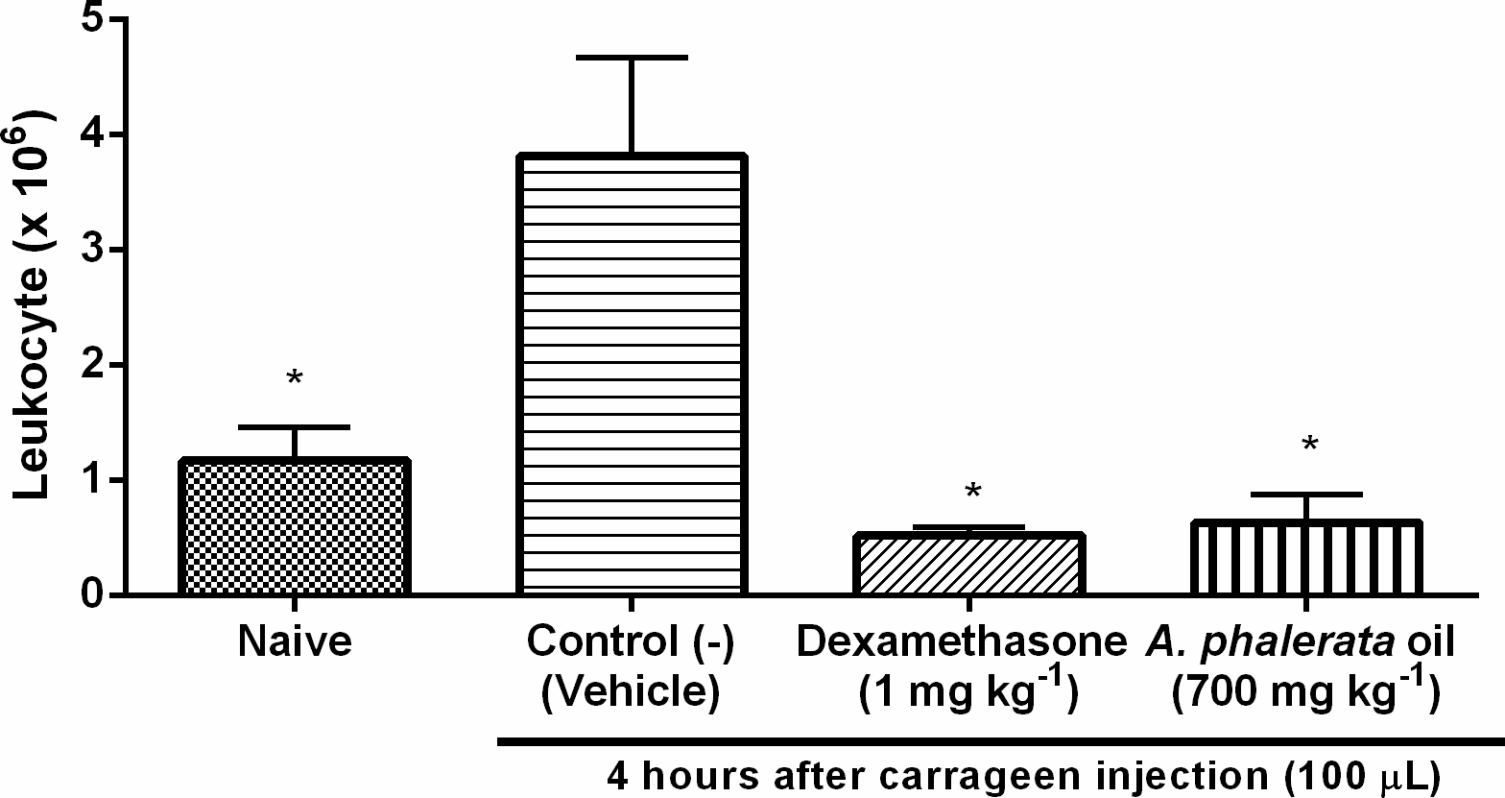
Effect of the *A*. *phalerata* oil on the leukocyte migration induced by carrageenan in the pleurisy model in rats. Values expressed in mean ± standard error of the mean. n = 5. * P <0.05 (ANOVA / Tukey) compared with the negative control (-).

The experimental model of Carrageenan-induced pleurisy has the ability to represent the main events of acute inflammation in a manner similar to that in humans; Thus, this model can be considered very efficient in investigating anti-inflammatory effects on potential substances [54]. Oral administration of APOP at a dose of 700 mg kg^-1^ significantly inhibited the inflammatory process (Fig 6), evidenced by the reduction in leukocyte migration to the pleural cavity. There was inhibition of 86 ± 4% of APOP in 4 h after administration of the phlogistic agent. The method evaluated only the leukocyte migration, without differentiation, but it can be assumed that the majority of the quantified cells are polymorphonucleated, with predominance of neutrophils. Neutrophils are the first cells to migrate to the inflammatory region and are present in greater amounts in acute inflammatory processes [55].

Thus, it is demonstrated that the compounds present in APOP have an action against the leukocyte migration, besides anti-edematogenic action, evidenced in the paw edema model. Our results corroborate with those presented by Batista et al. [56], which observed a decrease in the inflammatory process of tissue edema in the healing of cutaneous wound in rats after application of the oil of the pulp of *Caryocar coriaceum* Wittm, and Lescano et al. [57] that found anti-edematogenic activity and action against the leukocyte migration of the oil of the pulp of *Acrocomia aculeata* (Jacq.) Lodd. in experimental models of paw edema and pleurisy in *Wistar* rats, both attributing the action to the presence of carotenoids and unsaturated fatty acids.

### Antiproliferative and cytoprotective evaluation

The role of carotenoids on inflammation processes is directly associated with the inhibition of the generation of reactive oxygen and nitrogen species [58,59], that is, protection against cellular damage caused by oxidative stress [9]. Thus, it can be assumed that natural products, with high carotenoid content, have protective action in non-tumor cells exposed to chemotherapy. The results showed that APOP did not exhibit inhibitory action on cell proliferation in kidney carcinoma (786–0) and prostate carcinoma (PC-03) lineages, without demonstrating toxicity (Table 2).

**Table 2:** Effect of the *A*. *phalerata* oil in antiproliferative activity.

Compounds	IC_50_ μg mL^-1^		
	786–0^a^	PC-03^b^	NIH/3T3^c^
*A*. *phalerata* oil	> 250	> 250	> 250
β-carotene	> 250	> 250	> 250
Doxorubicin^d^	< 25	< 25	< 25

The results are expressed as μg mL^-1^ concentration of sample necessary to inhibit 50% of the proliferation cells.

^a^Kidney carcinoma

^b^Prostate carcinoma

^c^Murine fibroblast and

^d^Positive control.

**Fig 7:**
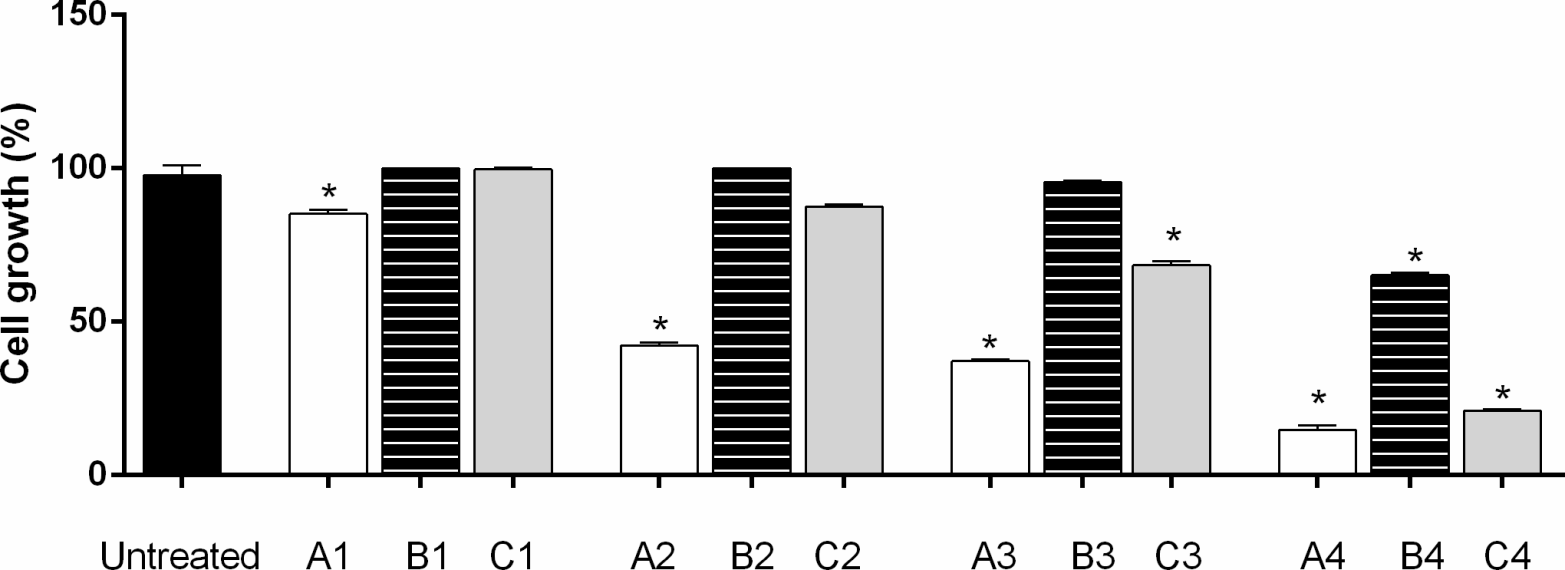
*In vitro* protective effect of *A*. *phalerata* oil under murine fibroblast cells (NIH / 3T3). The control represents untreated cells. A1, A2, A3 and A4 are evaluated treatment with doxorrubicina 0.025, 0.25, 2.5 and 25 μg mL^-1^, respectively. B1, B2, B3 and B4 are evaluated treatment with APOP 250, 500, 1000 and 2000 μg mL^-1^, respectively. C1-C4 are evaluated treatment combinations: (C1) used doxorrubicina on 0.025 μg mL^-1^ and *A*. *phalerata* oil on 250 μg mL^-1^; (C2) doxorrubicina on 0.25 μg mL^-1^ and *A*. *phalerata* oil on 500 μg mL^-1^; (C3) doxorrubicina on 2.5 μg mL^-1^ and *A*. *phalerata* oil on 1000 μg mL^-1^ (C4) doxorrubicina on 25 μg mL^-1^ and *A*. *phalerata* oil on 2000 μg mL^-1^. Values expressed in mean ± standard error of the mean. n = 3. * P <0.05 (ANOVA / Tukey) compared with the untreated cells.

This protective effect was observed during pre-treatment with APOP of non-tumor cells (NIH / 3T3—murine fibroblast) in different concentrations (250 to 2000 μg mL^-1^), following treatment with classic chemotherapeutic doxorubicin at concentrations of 0.025 to 25 μg mL^-1^ (Fig 7).

The effect of prior exposure to APOP suggests a significant protective effect on doxorubicin-treated murine fibroblast cells when compared to cell viability of non-oil exposed cells (Fig 7). The increasing combinations (C1, C2, C3, and C4) of doxorubicin and APOP were evaluated in association, and an oil-dependent protective effect on increasing concentrations of the chemotherapeutic agent (Fig 7, combinations C1-C3) was observed. However, in combination C4 the protective effect of the oil was proportionally lower, where we detected a low cell growth rate of 15 and 66% for cells treated alone with doxorubicin (A1, A2, A3 and A4) and APOP (B4), respectively, suggesting an oil toxicity at very high concentrations as observed in combination C4 and B4 (APOP 2000 μg mL^-1^).

## Conclusions

The fruit pulp oil of *Attalea phalerata* Mart. ex Spreng. in *in vitro* models of inflammation has action on the production of oxide nitric, inhibition of the COX1, COX2 enzymes and induces macrophage to spreading. In the *in vivo* models of inflammation, the oil performed anti-inflammatory activity, reducing paw edema during the action of carrageenan and positively influenced leukocyte migration by the pleurisy model induced by carrageenan. In the evaluation of the antiproliferative and cytoprotective activity *in vitro*, the oil did not influence the cellular growth and presented cytoprotective capacity at the evaluated low doses, inhibiting the cytotoxic action of doxorubicin in the studied cells.

The fruit of *A*. *phalerata* is a promising species as nutraceutical food and herbal medicine. The results demonstrate relevant beneficial pharmacological activity and still support popular use.

## Abbreviations

CEUA: Comissão de Ética no Uso de Animais
CONCEA: Conselho Nacional de Controle de Experimentação Animal
COX: cyclooxygenase
DMSO: dimethylsulfoxide
HPLC: high performance liquid chromatography
iNOS: nitric oxide synthase
LPS: lipopolysaccharide
MTT: (3-(4,5-dimethylthiazol-2yl)-2,5-diphenyl tetrazoline bromide)
APOP: *Attalea* phalerata oil pulp
NO: nitric oxide
PBS: phosphate buffered saline
PG2ɑ: prostaglandin
RNS: reactive nitrogen species
ROS: reactive oxygen species
SRB: sulforhodamine B
UFGD: Universidade Federal da Grande Dourados
